# Transcriptomic analysis of *Amaranthus retroflex* resistant to PPO-inhibitory herbicides

**DOI:** 10.1371/journal.pone.0288775

**Published:** 2023-08-24

**Authors:** Yulian Guo, Yu Wang, Xiangyun Zang, Chan Luo, Chunyan Huang, Keqiang Cong, Xiaotong Guo

**Affiliations:** Institute of Plant Protection, Heilongjiang Academy of Agricultural Sciences, Harbin, Heilongjiang Province, China; South China Agricultural University, CHINA

## Abstract

*Amaranthus retroflexus* L. is one of the malignant weeds which can cause a reduction in the soybean yield. We found a population of *A*. *retroflexus* (R-Q) resistant to fomesafen through the initial screening of whole-plant dose response bioassay in the research. The resistance index of the population (R-Q) was 183 times of the sensitive population (S-N). The resistant and sensitive populations were used as experimental materials in the paper. Strand-specific RNA-Seq analyses of R‒Q and S‒N populations obtained from herbicide-treated and mock-treated leaf samples after treatment were conducted to generate a full-length transcriptome database. We analyzed differentially expressed genes (DEGs) among the R-Q and S‒N *A*. *retroflexus* populations treated with recommended dose and mock-treated on the 1^st^ (24 h) and 3^rd^ (72 h) days to identify genes involved in fomesafen resistance. All 82,287 unigenes were annotated by Blastx search with E-value < 0.00001 from 7 databases. A total of 94,815 DEGs among the three group comparisons were identified. Two nuclear genes encoding PPO (*PPX1* and *PPX2*) and five unigenes belonging to the AP2-EREBP, GRAS, NAC, bHLH and bZIP families exhibited different expression patterns between individuals of S‒N and R-Q populations. The *A*. *retroflexus* transcriptome and specific transcription factor families which can respond to fomesafen in resistant and susceptible genotypes were reported in this paper. The *PPX1* and *PPX2* genes of the target enzyme were identified. The study establishes the foundation for future research and provides opportunities to manage resistant weeds better.

## Introduction

*A*. *retroflexus* L. is one of the problematic weeds in soybean field [1]. In recent years, *A*. *retroflexus* has caused ecological and economic impacts as an aggressive weed that decreases crop quality and yields in agricultural areas [2, 3]. Herbicides are currently used to control *A*. *retroflexus* L., while the persistent use results in rapid development of weed resistance [4]. The ability to rapidly evolve resistance is one of the reasons why *Amaranthus* species are the most studied weeds, as evidenced by high citation counts in current weed science literature [5]. Resistance to fomesafen was the first reported in 2014 in Brazil, and reported in China until 2017 [1]. More recently, the control effect of fomesafen on *A*. *retroflexus* decreased significantly in soybean fields in China.

Fomesafen, acifluorfen, oxyfluorfen belonging to diphenyl ether herbicides represent the major PPO-inhibiting herbicides. PPO inhibitors prevent protoporphyrinogen IX from being converted to protoporphyrin IX by plastid-localizing PPO enzyme (*PPX2*) [6, 7]. Protoporphyrinogen IX accumulates in the cytoplasm and induces singlet oxygen formation and damages cell membranes in the presence of light [8]. The resistance mechanisms to PPO inhibitors in *Amaranthus palmer* and *Amaranthus tuberculatus* have been reported in previous studies. The target site resistance mechanism mainly accounts for the codon deletion in the *PPX2* gene, causing the lack of a glycine residue at the site 210 (ΔG210) of the PPO [9–12]. It has been reported site 128 arginine (Arg) was substituted by glycine (Gly) and methionine (Met) in the PPO of *Amaranthus palmer* [13]. Although the molecular mechanism of resistance evolution in many *Amaranthus* populations has been confirmed, the mechanism has not yet elucidated in *A*. *retroflexus* populations resistant to PPO inhibitors. The specific genes conferring metabolic resistance remain unknown. This slow research process has limited progress towards our complete understanding of the genetic background of *A*. *retroflexus* and the molecular resistance mechanism of *A*. *retroflexus*.

Increased publicly available genomic resources of *A*. *retroflexus* can expand and deepen ecological genetic investigations. Transcriptomics provides abundant resources to elucidate the action of herbicides in different weed species and understand the weed genetics and biology [14–22]. As part of goal to expand *A*. *retroflexus* genome resources and reveal the genetic basis of diphenyl ether resistance, we reveal an annotated fully transcriptome and detect gene expression differences in selected herbicide resistant strains on this basis. The use of whole-genome expression profiling can provide an unbiased estimate of which biological processes are differentially affected by herbicides between selection lines, but it represents an exploratory assessment that may or may not identify candidate involved in adaptation [18]. Therefore, we can deepen understanding of the gene expression changes associated with direct and related changes following artificial selection for weed resistance by this technology. We report the sequencing transcriptome result of *A*. *retroflexus* with high-throughput RNA-seq technology in this paper. We also provide an abundant resource for *A*. *retroflexus* genomics, including the first transcriptome identification of *PPX1* and *PPX2* genes and transcription factors that respond to fomesafen treatment in *A*. *retroflexus* resistant and susceptible genotypes.

## Materials and methods

### Plant materials and growth

The *A*. *retroflexus* (R-Q) seeds were collected from Nenjiang County (48° 59′ 37″ N, 126° 1′ 51″ E), Heilongjiang Province, China, where fomesafen has been used for many years. The *A*. *retroflexus* populations have resistance to acifluorfen-sodium, fluoroglycofen-ethyl, fomesafen, imazethapyr, and lactofen, and they may be cross-resistant to other herbicides in the ALS inhibitor (B/2) and PPO inhibitor (E/14) groups [1]. A sensitive population of *A*. *retroflexus* (S‒N) was sampled from a noncultivated area in Hulin City (45° 46′ 44″ N, 132° 56′ 12″ E), Heilongjiang Province, China.

*Amaranthus retroflexus* L. seeds for each experiment were sown in rectangular plastic pots (volume = 1920 cm^3^) containing a 2:1 mixture of soil and vermiculite. Plants were grown in day/night (29/19°C) greenhouse with supplemental light by high efficiency tri-primary color natural light tubes programmed for a 16-hr photoperiod.

For herbicide treatments, the susceptible and resistant seedlings were sprayed with fomesafen (Hansencn 250 SL, Qingdao Hansheng Biotechnology Co., Ltd., China) when they were 7–9 cm tall. Fomesafen was applied at 0, 150, 300, 450 and 600 g a.i. ha^-1^ using a track sprayer equipped with an ASS-4 Automatic Pesticide Spraying System equipped with the 80° flat fan nozzle (Institute of Information Technology, Beijing Academy of Agricultural and Forestry Sciences, China) delivering 280 litres·ha^-1^ of water at 0.3 MPa. Three replicate pots were used per herbicide treatment and population. On the 1st, 2nd, 3rd, and 15th days after spraying, the leaves were collected to store for RNA extraction at -80°C as the sample.

### RNA extraction

The total RNA was quantified and the quality was assessed using an Agilent 2100 Bioanalyzer (Agilent, http://www.agilent.com). Poly(A) RNA was isolated from the total RNA using the oligo d(T) magnetic bead binding method and the Poly(A) PuristTM Kit (#AM1916; Ambion, now Life Technologies, http://www.lifetechnologies.com/uk/en/home/brands/ambion.html. Isolated poly(A) RNA was eluted with 20 μL of RNase-free water. All of the experiments were performed following the protocols included with the kits, and the RNA was stored at -20°C.

#### PacBio SMRTbell library preparation and sequencing

The Iso-seq template was prepared according to protocol provided for the Iso-seq template of subsequent system. The strand cDNA was synthesized from 800–1000 ng RNA referring to instructions of SMARTer PCR cDNA Synthesis Kit (#634926; Clontech, http://www.clontech.com). The cDNAs obtained was measured by an Agilent 2100 Bioanalyzer (Agilent, http://www.agilent.com) and Qubit HS (Life Technologies, USA). The templates were bound to SA-DNA polymerase and V2 primers using the DNA polymerase Binding Kit 2.0 (part #001-672-551). The complexes of templates and polymerase were bound to magnetic beads (part #100-125- 900) and transferred to a 96-well PCR plate for processing on a PACBIO RS using C2 sequencing reagents. Each library underwent SMRT sequencing using two SMRT cells. Subreads were filtered and subjected to CCS using the SMRT Analysis Server 2.2.0 (Pacific Biosciences of California, Inc).

#### PacBio data analysis

The PacBio raw reads generated from SMRT Sequencing on the Sequel System were analyzed to obtain reads of insert (ROI), read classification, and read clusters using Quvier and SMRT analysis programs. The isoform-level clustering algorithm ICE (Iterative Clustering for Error Correction) was used to improve consensus accuracy and the full-length consensus sequences was polished from ICE by Quiver. The postcorrection accuracy of full-length transcripts generated by this method was more than 99%. In the absence of a reference gene, cd-hit software was used to remove redundancy in clustered and error-corrected transcripts [23].

#### Functional annotation of PacBio isoforms

The functions of isoforms were analysed and annotated by Blastx, NCBI nonredundant (NR), NCBI nonredundant nucleotide (NT), KEGG, InterPro protein, Cluster of euKaryotic Orthologous Groups of proteins (KOG), Swiss-Prot protein and GO databases. InterProScan-5.16–55.0 was performed to map known protein domains to all isoforms and GO terms were obtained using InterPro2GO mapping for InterPro domains [24].

#### Differential expression analysis

We collected leaves of the R-Q and S-N populations (a total of 18 samples) treated with 300 g ai ha^-1^ fomesafen and the untreated control, respectively, on the 1st and 3rd days after spraying. Total RNA was extracted same as above and RNA sequencing (RNA-seq) was performed by the BGISEQ-500 platform at BGI (Shenzhen, China) [25, 26]. Sequence reads were mapped to the aforementioned PacBio isoforms (PRJNA929075) by Bowtie2 software (https://bowtie-bio.sourceforge.net/) and differential expression analysis of the two groups was performed using DESeq (https://www.bioconductor.org/packages//2.10/bioc/html/DESeq.html) [27, 28]. The fragment counts of each gene was normalized with fragments per kb per million (FPKM). The PacBio raw reads have been deposited in the NCBI Sequence Read Archive (SRA, https://www.ncbi.nlm.nih.gov/sra) database (PRJNA929075).

#### Differentially expressed gene analysis

Gene expression levels of each sample were estimated by RSEM as described previously [29]. The clean reads were mapped back onto assembled transcriptome and the read count of each gene from the mapping results were normalized as FPKM. Expression differences analysis of two groups were performed by DESeq and resulting Q-values were adjusted with approach described by Storey and Tibshirani to control the false discovery rate [30]. Gene expression differences were identified with an adjusted Q-value ≤ 0.001 (fold change ≥ 2) by DESeq.

Gene expression differences in R-Q and S‒N populations were submitted to Kyoto Encyclopedia of Genes and Genomes (KEGG) pathway analysis and Gene Ontology (GO) functional analysis. The open reading frame (ORF) of each unigene was identified by getorf (http://emboss.bioinformatics.nl/cgi-bin/emboss/getorf) and aligned to transcription factor (TF) domains with hmmsearch (http://hmmer.org/) [31].

#### Quantitative real-time PCR

Nine primers with variable or stable RNA-Seq expression patterns were designed through Primer3Plus S1 Table in S1 File [32]. The specificity of amplification and the effectiveness of qPCR were evaluated [33]. The each contig expression level of 26 original RNA samples were measured. The total RNA was extracted as above method and synthesized cDNA according to the instruction of PrimeScript™ RT reagent kit (Takara, Dalian, China). The qRT‒PCR system was consisted of 10 μM each primer (0.8 μL), RNase-free water (6 μL), SYBR Premix Ex Taq II (10 μL) (Takara, Dalian, China) and cDNA (2 μL). The reaction was performed on the ABI StepOnePlus Real-Time PCR System (Applied Biosystems, Foster City, CA, USA). The procedure of qRT‒PCR was composed of 95°C (30 s), 40 cycles of 95°C (5 s) and 60°C (30 s). Each experiment set three independent replicates. Error bars were calculated according to values of three replicates. Target genes relative expression levels were calculated through 2^-ΔΔCt^ method [34].

### Data access

The accession number of RNA-Seq data is PRJNA929075 in Gene Expression Omnibus (GEO, https://www.ncbi.nlm.nih.gov/geo/) database.

## Results

### Full-length transcriptome sequencing and assembly of *A*. *retroflexus*

Strand-specific RNA-Seq was applied to assess RNAs obtained from S‒N and R-Q populations to construct the full-length transcriptome database of this study and to comprehensively identify the unigenes associated with herbicide resistance. These mRNA samples from three S‒N and three R-Q populations leaf tissues of *A*. *retroflexus* were submitted to 2×100 paired-end sequencing with PacBio Sequel sequencer. Then full-length cDNAs from two pooled poly(A) RNA samples of R-Q and S‒N were normalized and submitted to SMRT sequencing by PacBio platform.

In total, 765,190 and 267,811 raw reads (22.05 and 8.35 GB bases) from the R-Q and S‒N populations, respectively, were generated by PacBio Sequel (Table 1). The 20,625,475 and 6,259,409 subreads from the R-Q and S‒N populations were filtered using SMRT analysis representing 18.00 and 7.30 GB bases respectively (Table 1). The 2 samples and 2 cells were sequenced using Pacific Bioscience Sequel platform, which included Classify and Cluster applications to generate CCS data. The more accurate sequence information was provided from reads through the insert at least three times [35]. In total, 1,325,392,600 bp of CCS (reads of insert) data (911,299 reads) were obtained.

**Table 1:** Summary of *A*. *retroflexus* transcriptome sequencing and assembly.

	Sample	
**Summary** ^1^	**S-N**	**R-N**
Total Raw Reads	765,190	267,811
Total Base (GB)	22.05	8.35
Total Subreads	20,625,475	6,259,409
Subreads Base (GB)	18.00	7.3
Reads of Insert^2^	667,095	244,204
Read Bases of Insert (bp)	950,056,548	375,336,052
Full-length Non-chimeric Reads^3^	285,632 (42.82%)	109,895 (45%)
Full-length Non-chimeric Read Length (bp)^4^	1,095.77	1,265.98
sLibrary genes of final consensus isoforms^5^	103806	45220
[All Samples] genes of final consensus isoforms	58190	24097

^1^The information of Polymerase Reads in raw data were summarized in Table 2 “Total Raw Reads” and “Total Base (GB)”. Detial information of Subreads in this project were summarized in “Total Subreads” and “Subreads Base (GB)”.

^2^They were classified to four categories including full-length non- chimeric, chimeric, non-full-lenth and short reads, by whether five primer, three primer and poly A were detected.

^3^Only the reads with two primer and poly A tail would be classified to full-length reads.

^4^Both full-length non-chimeric reads and non-full-length reads were used in further analysis.

^5^High quality consensus isoforms of each library would be merged into final result and redundancy would be removed.

**Fig 1:**
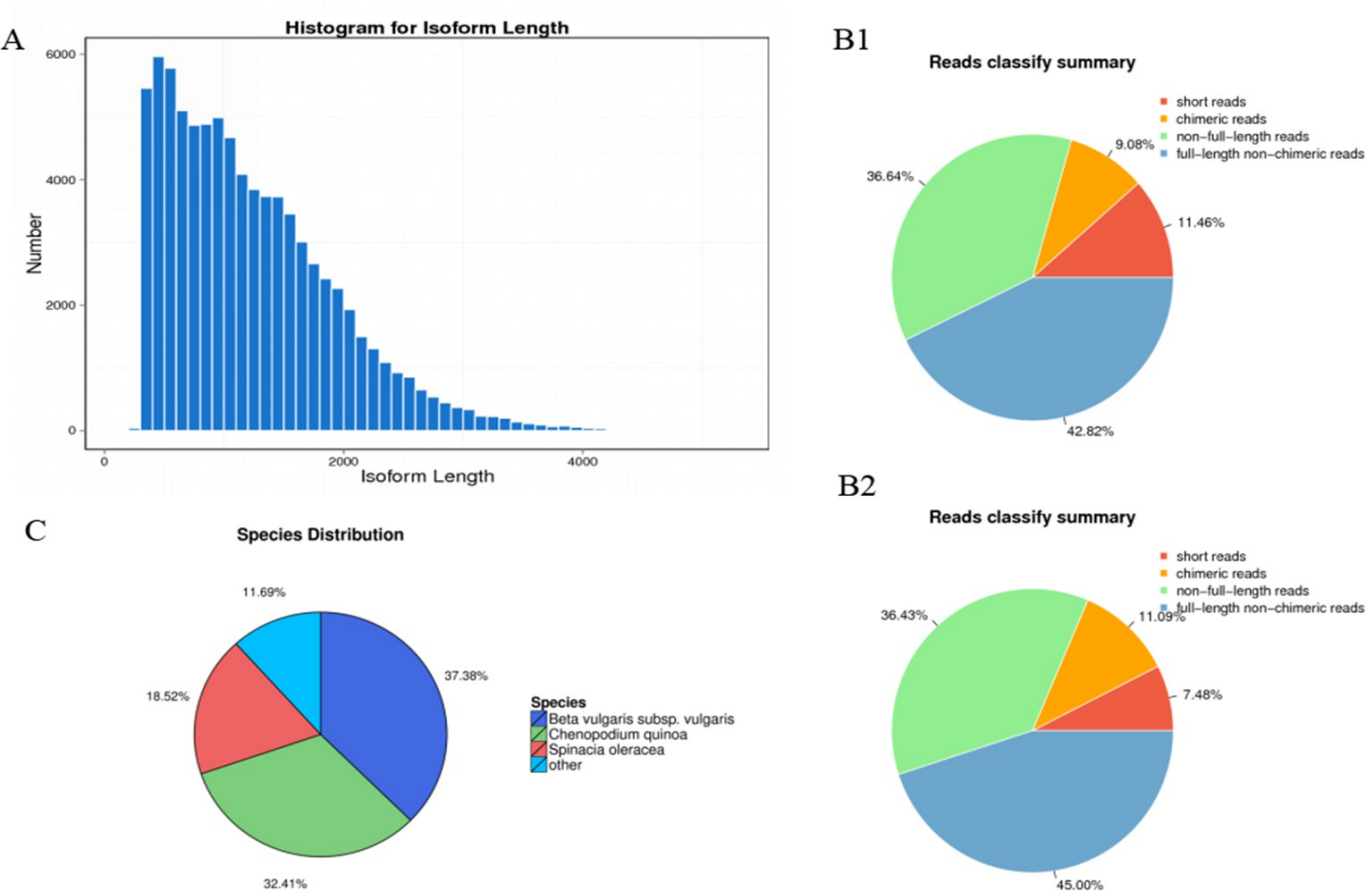
The detailed information of 285,632 and 109,895 full-length reads. (A) Length distribution of final consensus isoforms. Reads of insert were classified into four categories, including full-length nonchimeric, chimeric, non-full-length and short reads, based on whether five primers, three primers and poly A were detected. Only reads with two primers and poly A tail were classified as full-length reads. The pie chart in Fig 1B (B1:S‒N, B2: R-Q) illustrates the proportion of each category. (C) Distribution of NR-annotated species.

The 285,632 and 109,895 full-length reads were respectively obtained from the R-Q and S‒N by detecting the poly (A), the 5′ and 3′ primers and sequences in Table 1 and Fig 1. It confirmed the quality and breadth of our assembly compared to 113,893 transcripts of de novo waterhemp transcriptome [36] and 203,054 (>200 bp) transcripts from *Conyza bonariensis* transcriptome assembling produced [37]. Therefore, it provided the large scale and high-quality results in this paper that can serve as molecular reference for further *A*. *retroflexus* studies.

### Functional annotation

The 82,287 isoforms were obtained after cutting improper sequences, the total base and mean N50, and length of these unigenes was 100,787,516 bp, 1,225 bp and 1,534 bp, respectively (Table 1). The average contig size was 1,225 bp matching the average length of coding sequences in dicots (900 to 1400 bp) [38]. Regarding functional annotation, all 82,287 detected unigenes were annotated referred to Blastx searches from seven databases (Table 2). 73,040 (88.76%) transcripts were assigned initially Blast hits from the Nr database. Regarding top Blast hit species distribution of transcripts aligned in Nr database, 37.38% of genes were matched with sequences from *S*. *vulgaris* followed by *Chenopodium quinoa* (32.41%), *Spinacia oleracea* (13.75%), *Amaranthus tricolor* (0.95%), *Amaranthus hypochondriacus* (0.87%), *Amaranthus hybridus subsp*. *Cruentus* (0.11%), *Amaranthus retroflexus* (0.05%) and *Amaranthus tuberculatus* (0.04%) (Fig 1C). Few unigenes were *A*. *retroflexus* possibly because the limited data of the searches on *A*. *retroflexus* was contained in public databases.

**Table 2:** Summary of functional annotation.

Database	Number	Percentage
Total	82,287	100%
Nr	73,040	88.76%
Nt	65,509	79.61%
Swissprot	57,764	70.20%
KEGG	57,527	69.91%
KOG	57,674	70.09%
Interpro	61,606	74.87%
GO	19,298	23.45%
Intersection^1^	12,317	14.97%
Overall^2^	76,033	92.40%

^1^The number of Unigenes which annotated by all the 7 functional databases.

^2^The number of transcripts which be annotated with at least one functional database.

### Differentially expressed gene (DEG) analysis

**Fig 2:**
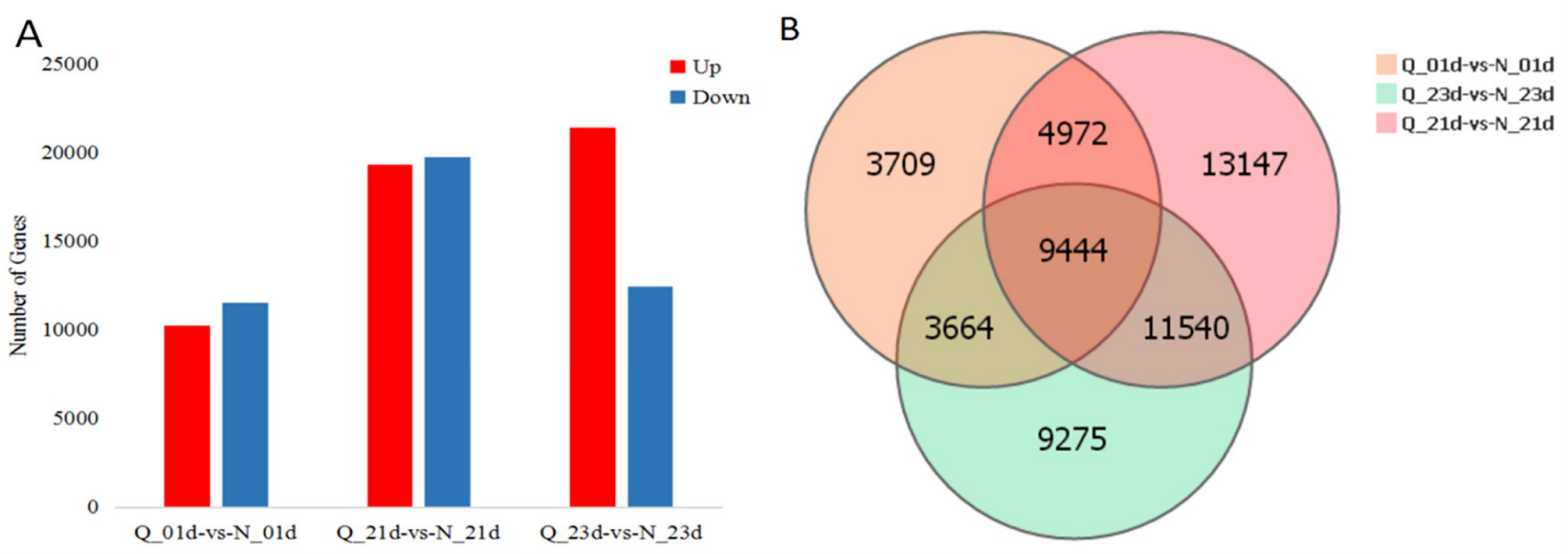
The analysis results of DEGs. (A) The number of DEGs among the Q-0-1day vs. N-0-1day, Q-2-1day vs. N-2-1day and Q-2-3day vs. N-2-3day groups are presented in histograms. (B) DEGs among the three group comparisons were identified using Venn diagrams.

The differences in resistance to fomesafen between S-N and R-Q *A*. *retroflexus* populations were screened at the genetic level, and DEGs among the resistant population (R-Q) and sensitive population (S‒N) under different treatments were analysed on different days. The genes in CK and 2^nd^ treatments were regarded as the background to identify DEGs on the 1^st^ and 3^rd^ days after spraying. In total, 21,789, 39,103 and 33,923 unigenes were differentially expressed in the Q-0-1day vs. N-0-1day, Q-2-1day vs. N-2-1day and Q-2-3day vs. N-2-3day groups, respectively (Table 3). In total, 10,258 upregulated DEGs and 11,531 downregulated DEGs were identified in the Q-0-1day vs. N-0-1day groups. The number of upregulated and downregulated DEGs were similar for the Q-2-1day vs. N-2-1day groups. Of note, the number of upregulated DEGs was abnormally increased compared with the number of downregulated DEGs in the Q-2-3day vs. N-2-3day groups with values of 21,453 and 12,470, respectively (Fig 2A). A total of 94,815 DEGs were identified using Venn diagrams of the three groups (Fig 2B).

**Table 3:** Quantity statistics of differential gene.

Compare Group	Up	Down	Total
N_01d^1^-vs-N_21d	17382	23140	40522
N_01d-vs-N_23d	15214	31469	46683
N_21d-vs-N_23d	5517	19795	25312
Q_01d^2^-vs-N_01d	10258	11531	21789
Q_01d-vs-Q_21d	11812	17320	29132
Q_01d-vs-Q_23d	12759	35977	48736
Q_21d-vs-N_21d	19320	19783	39103

^1^Susceptible (S‒N) population in the CK treatment served on the 1st day after spraying.

^2^Resistant (R-Q) population in the CK treatment served on the 1st day after spraying.

### Gene Ontology (GO) analysis

**Fig 3:**
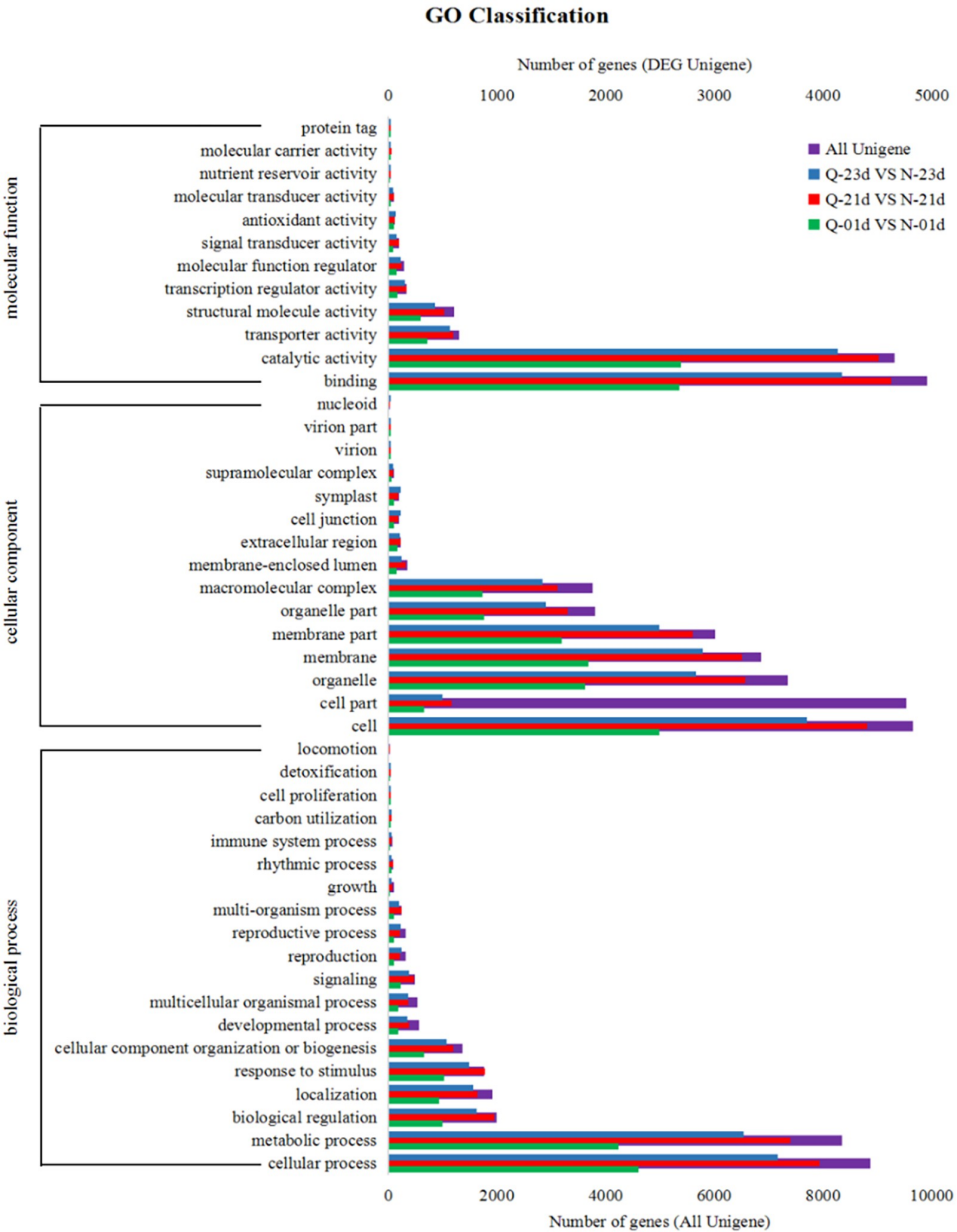
Summary of functional annotation results. Gene Ontology is divided into three functional classes: molecular function, cellular component and biological process. Functional classification was conducted according to the results of differential gene test. There are levels of subcategories under each category. The above figure shows the GO annotation classification results of differential genes. The X-axis represents the transcripts number and the Y axis represents the Gene Ontology function category.

The Gene Ontology (GO) analysis and KEGG pathway classification were performed to identify the function differences of these gene expression [39, 40]. In the GO classification analysis, 5266, 9052 and 8,095 unigenes from the Q-0-1day vs. N-0-1day, Q-2-1day vs. N-2-1day and Q-2-3day vs. N-2-3day groups, respectively, were classified to three main GO functional categories and divided into 47 subcategories. Many unigenes were assigned to more than one subcategory (Fig 3, S2 Table in S1 File). Among the Q-0-1day vs. N-0-1day, Q-2-1day vs. N-2-1day and Q-2-3day vs. N-2-3day group comparisons, 20 subcategories of biological processes were identified, and two of the largest subcategories were metabolic and cellular process. In total, 15 subcategories of cellular component category were identified, and the largest subcategory was “cell”, which contained 2,491; 4,399; and 3,851 unigenes for the Q-0-1day vs. N-0-1day, Q-2-1day vs. N-2-1day and Q-2-3day vs. N-2-3day group comparisons, respectively. In total, 12 GO subcategories of molecular function were identified, and the two largest subcategories included “binding” and “catalytic activity”. The results of GO functional classification analyses of DEGs were similar to those of all unigenes.

### KEGG pathway assignment analysis

**Fig 4:**
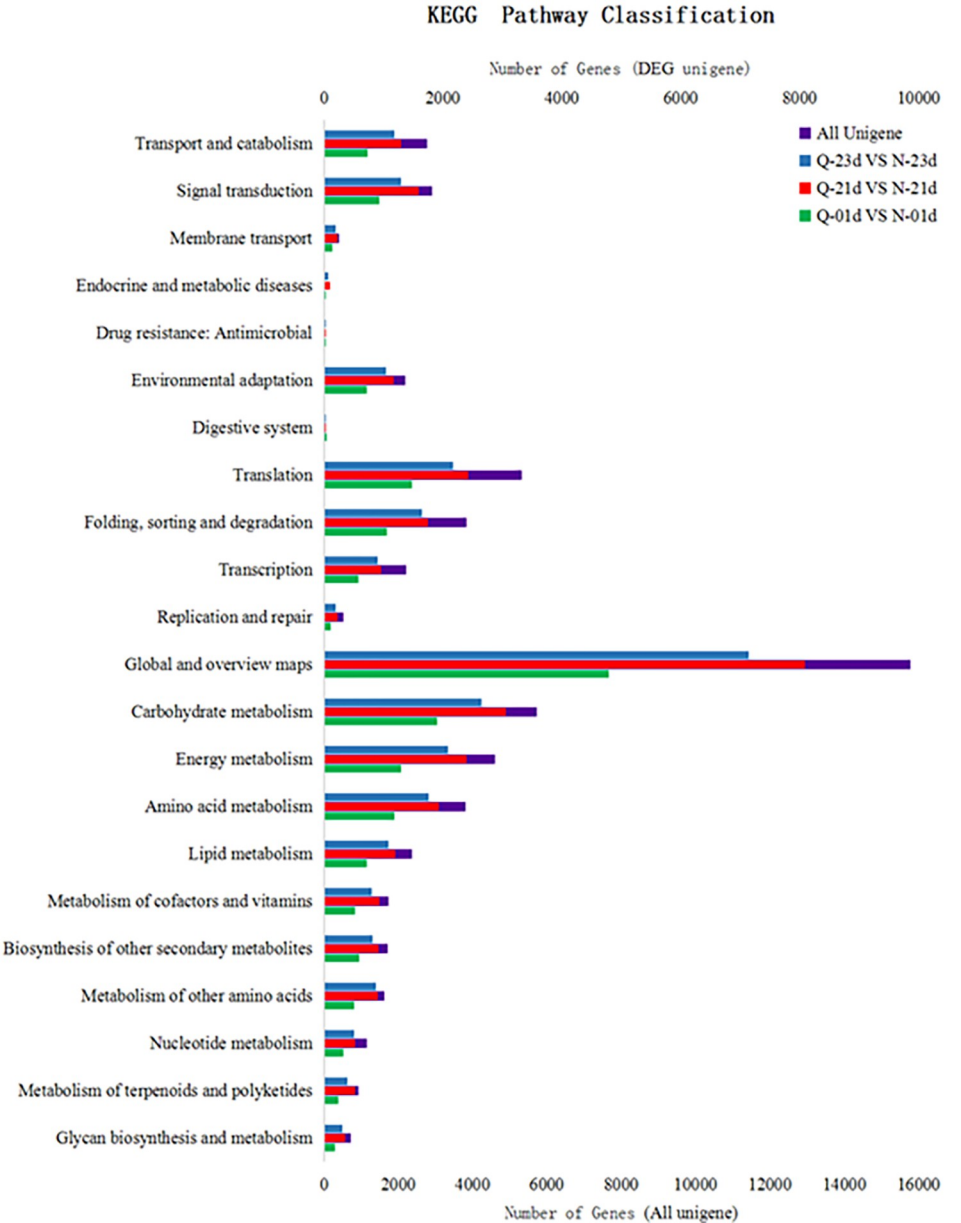
Functional distribution of KEGG annotation. The KEGG metabolic pathway involved in genes is divided into 7 branches: Cellular Processes, Environmental Information Processing, Genetic Information Processing, Human Disease (animals only), Metabolism, Organismal Systems and Drug Development. Statistics are further disaggregated under each branch. The image above is the KEGG Pathway annotated classification of the differential gene. The X-axis represents the transcripts number and the Y axis represents the KEGG function category.

The KEGG pathway assignment analysis showed that the DEGs were distributed across 22 KEGG categories (Fig 4). Among the Q-0-1day vs. N-0-1day, Q-2-1day vs. N-2-1day and Q-2-3day vs. N-2-3day group comparisons, the first category was “Metabolism”, which was divided into 11 subcategories, and two of the largest subcategories were “Carbohydrate metabolism” and “Global and overview maps”. The second category, “Genetic Information Processing”, contained 4 subcategories, and the largest subcategory was “Translation”. This category included 1,472; 2,412; and 2,162 unigenes for the Q-0-1day vs. N-0-1day, Q-2-1day vs. N-2-1day and Q-2-3day vs. N-2-3day group comparisons, respectively. The results of KEGG pathway analyses of DEGs were similar to those of all unigenes (Fig 4). When we concentrated on porphyrin and chlorophyll metabolism, 112, 205 and 164 DEG unigenes were found in the Q-0-1day vs. N-0-1day, Q-2-1day vs. N-2-1day and Q-2-3day vs. N-2-3day group comparisons respectively. These DEG unigenes were mapped onto “Global and overview maps” and “Metabolism of cofactors and vitamins” (S3 Table in S1 File) which can present exact and directed information to deeply analyze.

### qRT‒PCR analysis of functional genes

The primers were designed for Isoform_29331 (*PPX1*), Isoform_32632 (*PPX1*), Isoform_19725 (*PPX2*), Isoform_39222 (*PPX2*) and five genes of TF families (The AP2-EREBP, GRAS, NAC, bHLH and bZIP families were respectively represented by one gene Isoform_18616, Isoform_24299, Isoform_10392, Isoform_19481 and Isoform_5278) and performed real-time quantitative PCR analysis (Table 1).

**Fig 5:**
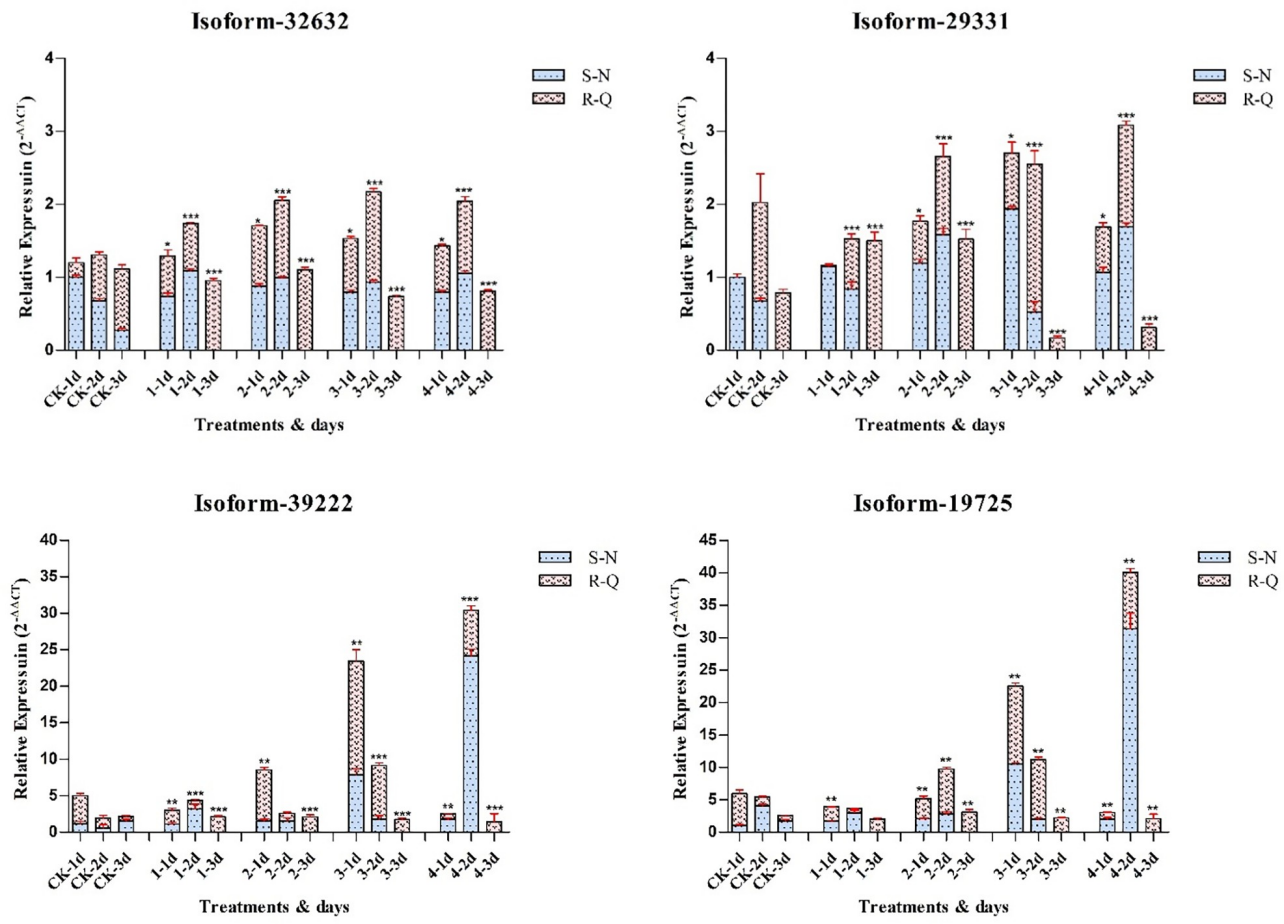
qRT‒PCR validations for the four genes showed relative expression in *A*. *retroflexus* samples. Dark bars indicate qRT‒PCR validation using the RNA-Seq samples of PPO herbicide-resistant (R-Q) populations, whereas red bars represent qRT‒PCR validation by the RNA-Seq samples of PPO herbicide-susceptible (S‒N) populations. The X-axis represents the sampels were divided by herbicide application concentration 0 (CK), 1st, 2nd, 3rd and 4th treatments served on the 1st day, 2nd and 3rd after spraying and the Y axis represents the relative expression (2^-∆∆CT^). Means and SDs from three biological replicates are shown. * p<0.05, ** p<0.01, *** p<0.001.

The qRT‒PCR analysis results of the *PPX1* and *PPX2* encoded by 4 genes denoted that these 4 unigenes were upregulated under high-concentration herbicide treatments in the R-Q population and S‒N population, and the expression was significantly upregulated after herbicide application compared to the 1^st^ and 3^rd^ days (Fig 5). In the S‒N population, the gene expression levels were increased compared to R-Q under the same conditions and Isoform_32632 (*PPX1*) expressed higher in all treatments (Fig 5).

**Fig 6:**
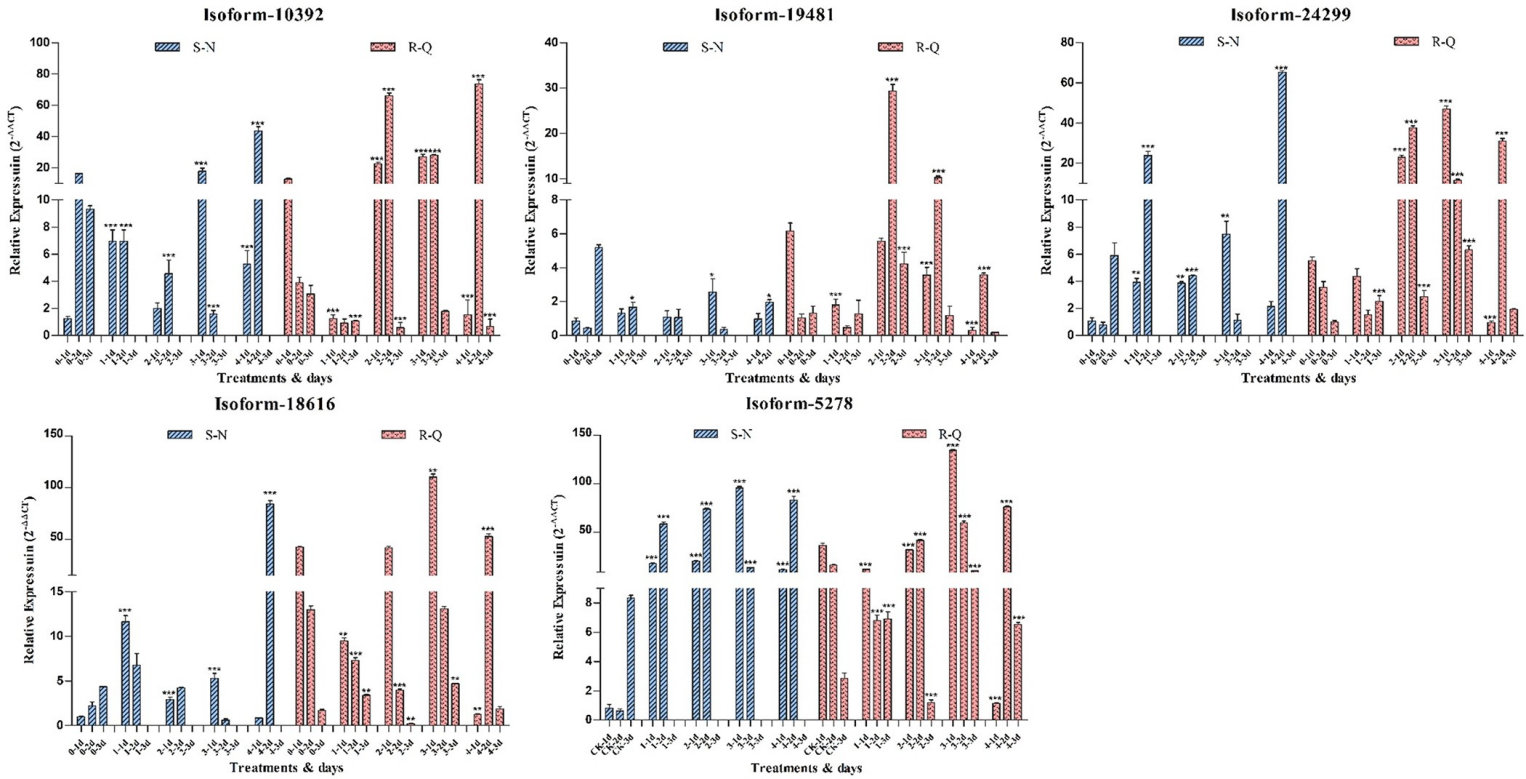
The qRT‒PCR validations for the five TF genes showed that relative expression in *A*. *retroflexus* samples. Dark bars denote the qRT‒PCR validation using the RNA-Seq samples of PPO-herbicide resistant (R-Q) populations and red bars present the qRT‒PCR validation using the RNA-Seq samples of PPO-herbicide susceptible (S‒N) populations. The X-axis represents the samples which are divided by herbicide application concentration 0 (CK), 1st, 2nd, 3rd and 4th treatments served on the 1st day, 2nd and 3rd after spraying and the Y axis represents the relative expression (2^-∆∆CT^). Means and SDs from three biological replicates are shown. * p<0.05, ** p<0.01, *** p<0.001. In the R-Q population, Isoform_24299, Isoform_5278 and Isoform_10392 (GRAS, bZIP and NAC1 family genes) expression exhibited similar trends (Fig 6). In the 0–3 treatments, except for the 1–2 d and 2–2 d samples, the expression level of the three genes in the other treatments was downregulated with time increasing. The expression level of Isoform_10392 was similar in the 3–1 d and 3–2 d samples. The expression of Isoform_24299, Isoform_19481 and Isoform_5278 (GARS, bHLH and bZIP family genes) was evidently lower in R-Q populations compared to S‒N populations in control check before two days, however it was opposite on the third day. In 1–2 d, these three genes were obviously upregulated in the S‒N population compared with the R-Q population (Fig 6). In R-Q, the expression of Isoform_24299, Isoform_19481, Isoform_10392, and Isoform_18616 (GARS, bHLH, NAC1 and AP2-EREBP family genes) increased rapidly and was significantly higher than that in S‒N on account of herbicide selective pressure (2 and 3 treatments). However, the expression of Isoform_5278 (bZIP family gene) was no differences between R-Q and S‒N populations under the same conditions (treatments 2 and 3) (Fig 6e). The interspecies comparison results indicated that herbicide selection pressure did not significantly affect Isoform_5278 gene expression between the R-Q and S‒N populations (Fig 6e). Notably, the expression of five genes was significantly upregulated only on the 2nd day during the whole treatment. These results demonstrated that these genes expression in the R-Q and S‒N populations was induced by high concentrations of herbicide.

A total of 14 candidate genes from the TF families were selected to screen genes associated with resistance. The qRT‒PCR was performed to confirm these 14 genes expression in 26 samples. Between the resistant (R-Q) and susceptible (S‒N) population, the samples from the CK and the 1^st^, 2^nd^, 3^rd^ and 4^th^ treatments served as a background were identified on the 1^st^, 2^nd^ and 3^rd^ days after spraying. The AP2-EREBP, GRAS, NAC, bHLH and bZIP families were each represented by one gene (Fig 6A–6E) (Isoform_18616, Isoform_24299, Isoform_10392, Isoform_19481 and Isoform_5278), and the remaining 9 genes were deemed to be false-positives.

## Discussion

*A*. *retroflexus* is an adatable herbicide-resistant weed found in agricultural areas of northeast China. The characteristics of *A*. *retroflexus* include growth rapidly, high reproduction coefficient, self-incompatibility and strong competitiveness with crop species. These characteristics boost the ability to evolve resistance, potential of crop yield losses and economic effects on agricultural production.

Several *Amaranthus* weed have been regarded as potential candidates for genomic studies [5, 41]. In this study, the resistant and susceptible *A*. *retroflexus* populations to fomesafen were selected. The sensitive *A*. *retroflexus* leaves were gradually damaged by fomesafen until the whole plant withered, whereas the resistant *A*. *retroflexus* population was able to resist fomesafen damage and continue to grow under the same concentration of fomesafen. PPO inhibitors prevent protoporphyrinogen IX from being converted to protoporphyrin IX by plastid-localizing PPO enzyme (*PPX2*) [6, 7]. Protoporphyrinogen IX accumulates in the cytoplasm and induces singlet oxygen formation and damages cell membranes in the presence of light [8]. However, the mechanism of *A*. *retroflexus* resistance to PPO inhibitors related to genes conferring metabolic resistance remains unknown. We employed genomic technologies to confirm genes and networks for *A*. *retroflexus* which are important for food security [42].

*Amaranthus* species has become the most studied weeds account for the ability to evolve resistance rapidly, as evidenced by the numerous citations in the current weed scientific literature [5]. More recently, failure of fomesafen to control *A*. *retroflexus* populations occurs frequently in soybean fields in China. However, genomic resources for studying traits of interest in *Amaranthus retroflexus* are limited. In this study, two multiplexed libraries were applied to de novo transcriptome assembly approach and sequenced to represent susceptible and resistant *A*. *retroflexus* genotypes treated with fomesafen over 48 hours. We built a compositive *A*. *retroflexus* transcriptome by the assembly of RNA-seq data in different libraries, which is a significant tool for scientific communities. Comparing gene expression between crop and weeds could present insights into novel targets for weed genes to control weed better. Researchers could study weed traits and provide perspectives on photosynthesis applied to crop improvement according to this information [36]. The original sequences have been preserved in the National Center for Biotechnology Small Reads archive (PRJNA929075), allowing for recombination and continuous improvement as sequences from *A*. *retroflexus* are available.

The *A*. *retroflexus* transcriptome was mined to demonstrate the application of these data for any transcript containing ‘porphyrin and chlorophyll metabolis’ in its annotation (S4 Table in S1 File). In the porphyrin and chlorophyll metabolism pathways, the PPO is the last key enzyme in pathway of haem and chlorophyll synthesis and oxidizes protoporphyrinogen IX (Protogen IX) to protoporphyrin IX (ProtoIX) [43]. When porphyrin and chlorophyll metabolism were studied, the 112, 205 and 164 DEG unigenes in the Q-0-1day vs. N-0-1day, Q-2-1day vs. N-2-1day and Q-2-3day vs. N-2-3day groups were identified. These DEG unigenes were involved in “Global and overview maps” and “Metabolism of cofactors and vitamins”.

In the full-length transcriptome database of this study, 86 unigenes involved in coding Protox (S5 Table in S1 File) were found. Among them, 10 DEG unigenes were identified between the S‒N and R-Q populations. Six unigenes exhibited greater than 91% homology, and BLAST alignment results indicated that these unigenes belonged to protoporphyrinogen oxidase (*PPX1*) mRNA. The other 4 unigenes exhibited greater than 97% homology, and BLAST alignment results indicated that these unigenes belonged to protoporphyrinogen oxidase (*PPX2* and *PPX2L*) (S6 Fig in S1 File). In plants, PPO was encoded by *PPX1* and *PPX2* [44, 45]. The proteins encoded by these genes have limited sequence identity (about 25%) and differ in subcellular localization [46–48]. The primers were designed and real-time quantitative PCR analysis was performed for Isoform_29331 (*PPX1*), Isoform_32632 (*PPX1*), Isoform_19725 (*PPX2*) and Isoform_39222 (*PPX2*) (Fig 5). The results of this study indicated that high concentrations of herbicides may induce *PPX1* and *PPX2* gene expression, and gene expression was associated with response time. Earlier studies suggested that the product of *PPX1* and *PPX2* can work in plastids and mitochondria respectively. Inhibition of PPO by herbicides can lead to the accumulation of protogen IX which is the substrate of PPO [7, 49, 50]. Protogen IX is transported from organelle to the cytoplasm and converted to proto IX by peroxidase. Proto IX induces the production of destructive singlet oxygen under the light, resulting in plant death [51, 52].

Transcription factors (TFs) play the key role almost in all biological processes [53]. Environmental stress to plants can cause a lot of transcriptional interference. The multiple stresses were adapted by activating and inhibiting the coordination of transcription factors on specific target genes [54]. The TF families were found, and 2450 unigenes were divided into 53 TF families in this study (S7 Fig in S1 File). The expression differences values of TF families (log2-fold change) between resistant and susceptible *A*. *retroflexus* were analyzed under the same conditions. The MYB, AP2-EREBP, GRAS, NAC, bHLH, bZIP and Sigma70-like (S8 Table in S1 File) families are related to the regulation of plant development and adversity responses, phytochrome signal transduction, light and stress signal transduction or control expression of groups of plastid genes [53–58].

Afterwards, a total of 14 candidate genes from the eight abovementioned TF families were selected to screen genes associated with resistance. The qRT‒PCR was performed to confirm these 14 genes expression in 26 samples. Between the resistant (R-Q) and susceptible (S‒N) population, the samples from the CK and the 1^st^, 2^nd^, 3^rd^ and 4^th^ treatments that served as a background were identified on the 1^st^, 2^nd^ and 3^rd^ days after spraying. The AP2-EREBP, GRAS, NAC, bHLH and bZIP families were each represented by one gene (Fig 6A–6E) (Isoform_18616, Isoform_24299, Isoform_10392, Isoform_19481 and Isoform_5278), and the remaining 9 genes were deemed to be false-positives.

Notably, the expression of these five genes was significantly upregulated on the 2nd day of the 4-day treatment compared with the 1^st^ and 3^rd^ days after herbicide application. These results demonstrated that these genes expression in the R-Q and S‒N populations was induced by high concentrations of herbicide stress. The light and stress signalling can regulated by basic region/leucine zipper motif (bZIP) transcription factors in plants [53]. Several reports indicated that NAC proteins have been involved in hormone-related processes during plant growth and stresses from environment [59–62]. The transcriptional regulation of phytochrome A signal transduction was facilitated by the GRAS gene family [55]. bHLH proteins from plants can work in transcriptional regulation associated with phytoch rome signalling [58]. AP2/EREBP transcription factors are one of the most conserved gene families and play an important role in plant growth and stress response [63]. In summary, these results suggest that these genes likely promote reproductive growth under the stress of high-concentration herbicide conditions in R-Q populations. This phenomenon constitutes an unknown resistance mechanism.

## Conclusions

Many important plant species to agriculture lack reference genomes. However, RNA-seq has provided scientific approaches to develop transcriptomes for characterizing genes and traits important for agronomic traits of both crops and weeds. The first *A*. *retroflexus* transcriptome was reported to expand genomics resources in this paper. The *PPX1*and *PPX2* genes in specific transcription factor families were identified and given the roles in responding to PPO inhibitors in resistant and susceptible genotypes. The results will serve as a data resource for further studies on the molecular mechanisms of resistant *A*. *retroflexus* to diphenyl ether herbicides. Further studies will involve functional genomic analysis using the protoplasts and gene editing approaches.

## Supporting information

S1 File (RAR)
